# Medicine and health of 21st Century: Not just a high biotech-driven solution

**DOI:** 10.1038/s41525-022-00336-7

**Published:** 2022-11-15

**Authors:** Mourad Assidi, Abdelbaset Buhmeida, Bruce Budowle

**Affiliations:** 1 Center of Excellence in Genomic Medicine Research, King Abdulaziz University, Jeddah, Saudi Arabia; 2 Medical Laboratory Department, Faculty of Applied Medical Sciences, King Abdulaziz University, Jeddah, Saudi Arabia; 3 Department of Forensic Medicine, University of Helsinki, Helsinki, Finland

**Keywords:** Quality of life, Molecular medicine

## Abstract

Many biotechnological innovations have shaped the contemporary healthcare system (CHS) with significant progress to treat or cure several acute conditions and diseases of known causes (particularly infectious, trauma). Some have been successful while others have created additional health care challenges. For example, a reliance on drugs has not been a panacea to meet the challenges related to multifactorial noncommunicable diseases (NCDs)—the main health burden of the 21st century. In contrast, the advent of omics-based and big data technologies has raised global hope to predict, treat, and/or cure NCDs, effectively fight even the current COVID-19 pandemic, and improve overall healthcare outcomes. Although this digital revolution has introduced extensive changes on all aspects of contemporary society, economy, firms, job market, and healthcare management, it is facing and will face several intrinsic and extrinsic challenges, impacting precision medicine implementation, costs, possible outcomes, and managing expectations. With all of biotechnology’s exciting promises, biological systems’ complexity, unfortunately, continues to be underestimated since it cannot readily be compartmentalized as an independent and segregated set of problems, and therefore is, in a number of situations, not readily mimicable by the current algorithm-building proficiency tools. Although the potential of biotechnology is motivating, we should not lose sight of approaches that may not seem as glamorous but can have large impacts on the healthcare of many and across disparate population groups. A balanced approach of “omics and big data” solution in CHS along with a large scale, simpler, and suitable strategies should be defined with expectations properly managed.

## Historical context of over-reliance on biotechnology-driven treatments

The contemporary healthcare system (CHS) has been shaped by century-long innovations and discoveries made notably in the late 1800s and early 1900s^1^. To achieve the ultimate goal of allowing people to live longer and healthier, scientists and clinicians, among others, have made remarkable efforts to continuously enhance the CHS, which has improved the lives of nearly every person on the planet, although not necessarily equally (i.e., there are disparity issues that need to be addressed). While preventive healthcare is practiced by some, CHS is mainly a reactive approach strategy that often waits until the person becomes ill with acute symptoms to undertake a specific surgical intervention and/or a drug-based corrective action.

### The “magic bullet” era

Drug therapy began centuries ago with the use of plant extracts and progressively evolved into the development of purified and targeted materials for a wide range of health-related applications, such as morphine (1803), anesthetics (1840s), antipyretics, and analgesics (1870s) in the 19th century. At the beginning of the 20th century, Ehrlich’s research laid the foundations for drug screening and discovery by bridging the gap between chemistry, biology, and medicine. His research discovered one of the first “magic bullets”—the antisyphilitic drug Arsphenamine. Other treatments for other diseases were subsequently discovered, including insulin, penicillin, and chemotherapy^2,3^. Pharmacological research expanded significantly to develop new cures and ameliorative approaches for various diseases with many noted successes. Consequently, the proportion of patents and newly-developed pharmaceutical products increased from 25% of all pharmacy remedies in the 1940s to nearly 90% at the end of 20th century^2^. Based on the realized health benefits of drug therapy, there has become an enormous reliance on drug prescriptions and unprecedented levels of use^4^. With the rapid economic development and enhanced living standards since the end of World War II, the use of drugs has steadily increased, boosted in part by the support of insurance and social security systems.

Through marketing and lobbying adopted by pharmaceutical companies to continuously expand their markets and benefits^5^, a major bias has taken root in the public and healthcare providers’ mindset and culture: treatment of disease primarily is achieved through prescription of drugs with a concomitant (and unfortunate) lower reliance on prevention and health promotion^6,7^. This reliance on drug therapies has made their use varied and commonplace^4^, although some treatments have become cost prohibitive for the majority of the population contributing to healthcare disparity. The pushing of drug therapy without an appreciation of the human factor has seen a concomitant increase in patients suffering medication (ab)use and/or iatrogenic effects. Many individuals over 65 years of age in the Western world take anywhere between 5 to more than 20 drugs per day^4,8,9^. Moreover, most of these drugs are palliative treatments; for example, in the USA, 9 out of 10 prescribed drugs are pain killers and symptom relievers^10^. This drug reliance strategy has been associated with unprecedented waste in annual global healthcare expenditures due to overspending, unnecessary prescriptions, mistakes, and corruption costing upwards of USD 300 billion (according to the European Healthcare Fraud and Corruption Network)^11^. The epidemic opioids crisis in the USA is an example of drug (ab)use due to the underestimation of the neurobiological harm and the potential addiction effects mainly on individuals/groups with particular social vulnerabilities^12,13^. This crisis is a heavy public health burden that begets severe health, socioeconomic and legal consequences^12^.

### Other challenges of the “drug-only” solution

This “drug solution” problem, in turn, is exacerbated with new challenges related to availability, accessibility, affordability, safety and effectiveness^11^. Beyond the heavy financial burden of healthcare commodification and, more importantly, drug interactions and side effects—due to both polypharmacy and inappropriate prescriptions—have led to frailty, severe comorbidities, higher hospital admissions, and increased mortality^14^. Similar issues can be seen with other hopeful cures such as vaccine access to immunize the population suffering from the current pandemic. While rather elegant biotechnology-based solutions have been undertaken to rapidly develop SARS-CoV-2 vaccines (experiencing the fastest development of an approved vaccine(s) in history), the roll out and access to the vaccine to all population groups, as well as willingness by some to receive it, has been far more challenging^15–17^. One would have thought that the logistics for dissemination could have been planned better^18,19^. Innovation and cost of vaccine purchase were not impediments but instead, a more basic distribution strategy(ies) and information dissemination should have been implemented. A well-planned distribution strategy reduces the virus reservoir, impacts the greater population, and reduces health disparities.

Despite the considerable budget allocated to drug discovery, pharmacogenomics, and high biotechnology, these fields have substantial bottlenecks in CHS, as they have high rates of failure. These failures were in part due to instrumentation, methods, statistics, computational power, machine learning, etc. that were not able to accommodate, organize, and process the information needed to provide more precise solutions. In the USA, 90% of new drug applications to the FDA are rejected because of a lack of efficacy and/or toxicity^20^. Moreover, among the most prescribed drugs in the USA, the most successful one was reported to be effective in only 25% of patients^21^. This “imprecision medicine” is mostly due to the complexity of human biology systems, inappropriate or limited settings during, for example, the drug development process, and/or the inability of a specific drug to fix multi-level molecular perturbations. Omics solutions will help in predicting those patients in which a positive effect will occur and which patients who will have no effect or adverse reactions to the treatment. Because one can foresee drug development will be targeted to only those individuals with a positive effect, the cost will continue to rise, and likely health disparity will be further exacerbated.

## Advancements in biotechnology to improve human health

### The promise of omics and big data sciences

At the beginning of the 21st century, the completion of the human genome project (HGP) provided a blueprint map towards precision medicine (PM) with a promise to improve quality of life. The first deciphered blueprint in itself had little impact. However, the HGP fostered biotechnology innovation and advances in bioinformatics, such as exquisite massively parallel sequencing technologies turning the herculean effort of sequencing an entire human genome into a reasonable cost and trivial exercise today. Boosted by digital analytics, the HGP has metamorphosed the way life science research is conceived and applied. Subsequently, several new disciplines have emerged, such as biobanking, bioinformatics, comparative genomics, pharmacogenomics, clinical genomics, and projects such as the human proteome project, the human microbiome project, the cancer genome atlas project, and the illuminating druggable genome program, to name a few. Furthermore, the emergence of the digital revolution has been progressively introducing extensive changes on all aspects of contemporary society, economy, firms, and job market^22^. This huge impact has also encompassed the way science and research are conducted in every discipline^23^. In medicine, mega sets of sequence and metagenomic data, super-libraries of medical images, and complex drug databases are generated on a massive scale. These huge data sets are clear illustrations of a new complex, automated and data-driven trend to gain insights about both the clinical profiles and molecular signatures in health and disease statuses^24–26^. Experts estimate that innovations, such as omics biotechnologies mainly integrative personal omics profile (iPOP)^27^, connected health systems, wireless wearable devices, blockchain technology, the Internet of Things (IoT), health tokens, artificial intelligence (AI), and machine learning (ML) are promising ways to address CHS’s challenges^28–30^ (Table 1).

**Table 1:** An overview of the main recent innovations and advancements in biotechnologies and their potential applications to improve human health.

Technology/Innovation	Approximate date	Applications/benefits	Reference(s)
Recombinant DNA technology	1970s	Allowed experimental manipulation of DNA fragments in laboratory setting.	^74,75^
Monoclonal antibodies	1973	Mainly used in clinical setting for targeted therapy and prevention of transplant rejection.	^76,77^
RNA Interference	1990	This epigenetic process allowed post-transcriptional silencing of several pathogenic/unwanted genes in a range of organisms and foods.	^78–80^
Targeted therapy	1992	Allowed a more specific treatment of diseases using monoclonal antibodies.	
Human Genome Project	2003	Deciphering the human genome sequence and promoting a plethora of medical and non-medical applications.	^81–83^
Whole genome sequencing (NGS)	2005	Massively parallel sequencing technology generated huge amounts of DNA sequence data with higher accuracy that can be aligned and compared to a reference genome/sequence.	^84–86^
induced pluripotent stem cells (iPSCs)	2006	Provision of unprecedented opportunities for cell therapies against several diseases and injuries.	^87,88^
Artificial intelligence (AI) and machine learning (ML)	2010	These advanced smart computing technologies are providing substantial support to healthcare management processes, clinical decisions, robotic surgery, data archiving and sharing, as well as digital health.	^89–92^
Gene editing and gene therapy	2012	Site-specific editing/correction of DNA structure for therapeutic purposes.	^93,94^

With the advent of these omics-based biotechnologies (e.g., genomics, transcriptomics, epigenomics, proteomics, and metabolomics) and big data science, a new wave of hope has spread over the scientific and clinical communities as well as the general public, in search of instant, individualized, and accurate theranostics^31^. There are and will be CHS improvements at both the individual and population levels in NCDs and infectious diseases. While omics undoubtedly will impact positively precision medicine^32^, it is important not to lose focus that individualized solutions that can be leveraged to affect population level challenges still will provide the greatest improvement in CHS. One of the main outcomes of deciphering cancer using omics technologies was targeted therapies. In fact, targeted anticancer therapy (TAT) is an expanding area that revolutionized cancer treatment modalities and significantly improved prognosis, treatment, and prediction of several malignancies^33,34^. Furthermore, TATs have played a major role in converting several cancers from fatal diseases to manageable chronic conditions^35–38^. However, these TATs-induced improvements were lacking enough specificity and effectiveness. The wide genomic instability and tumor heterogeneity marked by myriad possible multi-mutations precludes any hope for precision treatments^33^. Therefore, TATs were often combined with the other treatment modalities as surgery, chemo, radio, hormonal therapy, and even other targeted therapies. So far, the developed TATs were not able to overcome the toxicity, and cross-reactivity on nontarget tissues, relapse, and drug resistance^37^. Notably, only a small proportion of the population benefits from TATs at a higher cost. Therefore, it is obvious that a “magic bullet” solution for cancer treatment is still unreachable.

Noteworthy, of five health determinants (genome and biology, lifestyle choices, social circumstances, environment, and healthcare system), medical care’s contribution does not exceed 11% of each individual’s health^39,40^. This means that 89% of one’s health is impacted by determinants outside of the CHS realm. Thus, more emphasis on the remaining health determinants will substantially improve CHS’s performance. A tendency to marginalize prevention and health promotion—perhaps due to its unprofitability character or its lack of glamour or lack of insurance support—has impeded more focus on implementation of health quality pillars and adequate prevention strategies.

For instance, half of all deaths in the U.S. were due to behavioral causes^41^ and therefore may be preventable. These health-related behaviors, which are only part of the problem, were mainly driven/influenced by social determinants as education, employment, and income^41,42^. Another illustration of the cost-effective and global impact of the health determinants outside the healthcare realm were the findings of McKeown who demonstrated that the sharp increases in life expectancy at the 19th century in the UK was mainly triggered by the improved living conditions, such as nutrition, sanitation, and potable water availability, decades ahead of the discovery of antibiotics, vaccines, and intensive care units^41,43^. Strikingly, more than 75% of healthcare spending in rich countries is dedicated to managing lifestyle-induced conditions. However, it is estimated that 80% of these NCDs are preventable by readily and cost-effective lifestyle choices improvements^44^. Taken together, these findings highlight that CHS effectiveness could not be enhanced by high-tech-driven inputs only but must consider the other health determinants as the foundation of any future reform.

Although better insights and resolution about diseases’ diagnoses and stratification, as well as healthcare management, can be observed given their descriptive character^45^, the digital revolution impact on precision therapeutics may not be realized readily, except for applications such as rapid vaccine development, robotic surgery, detection of unknown pathogens, disease monitoring and predicting adverse drug reactions to name a few. The new and unprecedented challenges are related to big data and biospecimens’ collection, storage, sharing, analysis, reliability, reproducibility, interpretation, governance, and bioethics that have emerged, with accompanying logistics requirements and considerations^46–49^. The PM concept at this post-genomic era—although inspirational—remains costly with limited success for population level impact at least in the short and medium term^50^. We are not advocating a reduced effort in this regard but managing expectations should become part of the strategy and more so not to lose sight of alternate not as “newsworthy” strategies that may have greater outreach to improving healthcare disparity and quality.

### Biology: inconceivable complexity nevertheless user-friendly

Human biology is a multi-layered complexity of dynamic and interactive networks at the single-cell, multicellular, tissue, organ, system, organismal, environmental as well as social levels. In this context, NCDs are a series of perturbations of afore described complex networks that are deeply rooted in the biology, lifestyle choices, and the engineered/devised environment in which we live today. Given its appearance as user-friendly, biology complexity continues to be, unfortunately, underestimated. While the HGP and the ensuing development of omics solutions have lofty goals, the problem of molecular complexity has been underestimated, and deciphering the genotype-phenotype relationship continues to plague reaching the “magic bullet” goal^51^. For example, Singh and Gupta point out that the unanticipated necessary and unnecessary complexity of molecular machinery and systems in conjunction with evolutionary processes make it extremely difficult, currently, to apply PM effectively. An organism’s genetic redundancy and multiple molecular pathways are complex, related, and integrated and they also affect traits and thus complicate interpretation. It should not be surprising that individuals with similar risk factors for a disease may have different phenotypes^52,53^. Genetic backgrounds, gene interaction networks, environments, and histories impact PM making it “uncertain, chance-ridden, and probabilistic”^52^.

The scientific community should be aware that these biological systems could not be compartmentalized as independent and segregated problems in the digital and molecular realm, and therefore are very challenging to be mimicable by the current digital tools. Although impressive strides have been made with the advent of customized artificial intelligence (AI) and machine learning (ML) algorithms that analyze the complexity of these still poorly understood biological networks, they likely will not achieve the status of the “magic bullet” solution in the near future. There is a need for education and training of algorithm-building proficiency experts—a fundamental part of the roll out of advance technological solutions that has not been a major focus of national or global strategies. Perhaps computer science or better yet bioinformatics should become a requisite course in the secondary school system or at least part of an undergraduate curriculum for all students.

It is noticeable that the development of technology and data-driven applications is significantly faster than the progress of the scientific understanding of the complex interactions in biology (e.g., assessing differences between association and causality) and related fields. The realization of AI, ML and big data promises to deliver accurate clinical decisions and robust therapeutic predictions will require first overcoming the major intrinsic challenges related to their origin and cause such as the 4 Vs (volume, velocity, variety, and veracity)^49,54,55^. The combination of complexity and limitations of big data and omics-based sciences, confounded with health disparities, has revealed that what we dream of as “precision medicine” is still “imprecision medicine”^56^. Beyond these complexities that will be challenges for the foreseeable future is the impediment of the rapidly developing, rapidly changing technology and bioinformatics. These dynamic changes are welcomed because they bring bigger and better ways to identify and use diagnostic and prognostic bioindicators. However, the swift change that is occurring is an indication that the -omics biotechnologies are far from mature and obviously not stable. Technology will have to become somewhat standardized (or stabilized) to differentiate variation in assay performance and noise from -omics contributions and to be able to compare data effectively among the multitude of studies. Moreover, unstable biotechnologies are challenging to implement into operation-oriented diagnostic laboratories as it is costly to invest, requires ever changing quality assurance practices, can create a chaotic environment and the staff in such facilities are users, not innovators of new technologies and will not be able to adjust and troubleshoot as problems arise. While there no doubt will be successes in healthcare developed in the post-genomic era, the belief that “omics and big data” are poised to become routine parts of the HCS may be premature.

## Population health and prevention: Simpler solutions matter

This high reliance on drugs has been a landmark that shaped the CHS. Unfortunately, this drug-only strategy was strongly applied to be the cure of multifactorial NCDs (e.g., cardiovascular diseases, cancer, chronic obstructive pulmonary diseases, diabetes, obesity, etc.), the current main global health burden. However, NCDs have complex causes. Their vectors are embedded in multi-generic effects as patients’ genetics (to include genetic imprinting), socio-economic environment, biology, and lifestyle choices^57,58^. Socio-economic vectors, a large and often under considered set of factors, for instance, encompass the complex interactions and disparities between economic growth, urbanization, aging, education, globalization, and the pervasiveness of unhealthy products on the market^59–61^. Given such inherent complexity, trying to develop drugs for NCDs using a reductionist approach likely will have limited positive results which at best will serve subcomponents of the population and may not have the global impact desired. The treatment concept for these diseases should move from a drug only strategy carapace to a more comprehensive approach of positive change/intervention in the individual/population socio-economic environment and lifestyle choices following a more holistic approach.

**Fig. 1:**
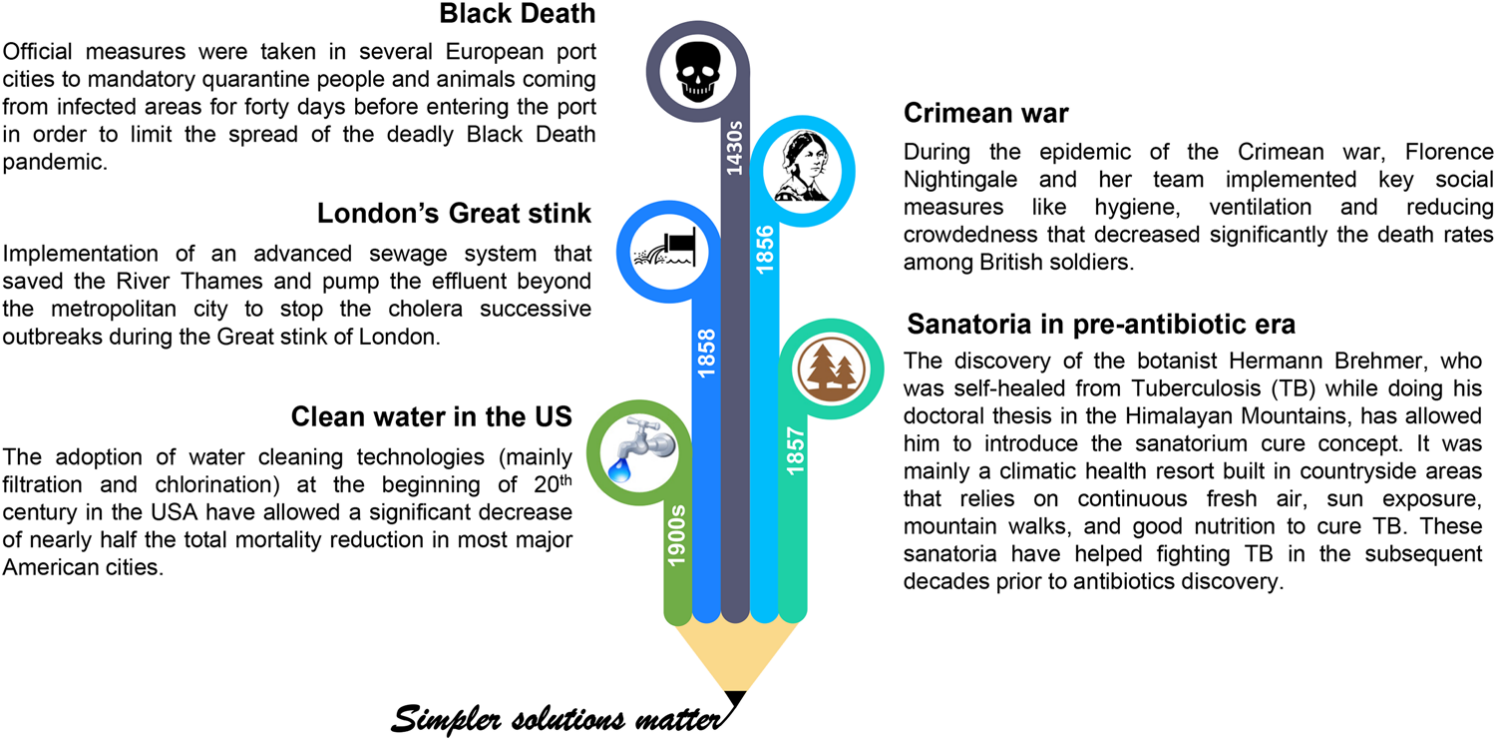
Some notable public measures outside the medical care realm that have significantly improved population health. These measures included quarantine during Black Death epidemic^95^, hygiene and social distancing in the Crimean war^62^, the building of mountain sanatoria to cure TB^96–98^, the implementation of a sewage system to overcome London’s Great stink^63,64^, and the introduction of water filtration and chlorination systems/technologies to clean potable water in the USA^99^.

History is replete of simpler solutions with large impact (Fig. 1). For example, during the Crimean war in 1854 where there was a shortage of medicines’ supply, the famous British nurse Florence Nightingale significantly reduced the death rates of wounded soldiers from 42 to 2%, and prevented mass infections mainly through improving the hygiene through hand washing, proper ventilation, reducing crowdedness, and sewage evacuation^62^ (Fig. 1). Also, and following three successive and unexplained tragic outbreaks of Cholera in London, the sewage system proposed by civil engineer Joseph Bazalgette in 1859, suggested by some historians as a “hero of London”, was able to stop the water-borne transmission of disease. The role of the implemented sewage system was to pump the effluent through several interconnecting pipes beyond the metropolitan city^63,64^. These examples, among others, clearly pinpoint that simpler and affordable measures outside the realm of HCS and advanced biotechnologies could have significant and sustainable impact on human quality of life, wellbeing, and sustainability.

## COVID-19 and Socio-Economic Disparities

**Fig. 2:**
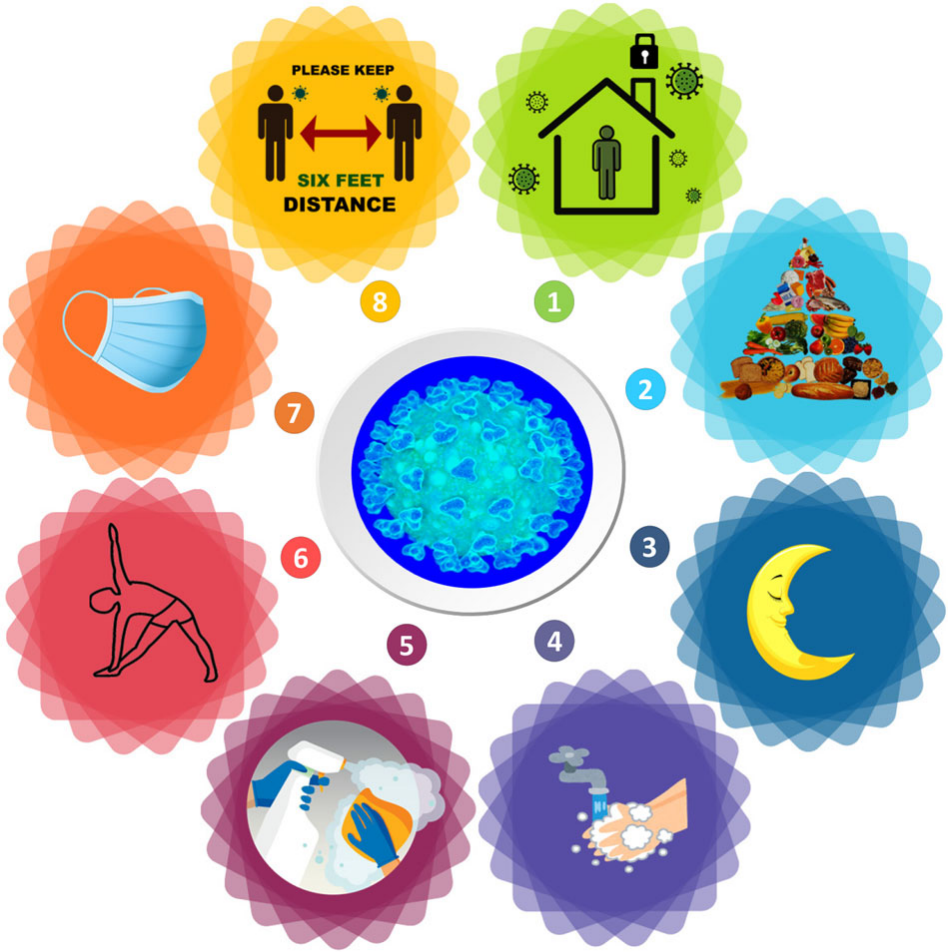
Main social measures that mitigated COVID-19 spread and helped in disease prevention and control (flatten the contagion curve) before vaccine development. (1: quarantine/lockdown/curfew; 2: healthy and balanced diet; 3: adequate sleeping; 4: frequent hand washing; 5: cleaning and disinfection of both surfaces and the air; 6: regular domestic exercise; 7: facemask wearing; and 8: social distancing).

Currently, for instance, the world is facing successive waves of the life-threatening COVID-19 pandemic. Sadly, this coronavirus has affected about a half billion people and killed over 6 million according to WHO^65^. Indeed, humanity is expressing a deep need for science to develop a “cure” with drugs/vaccines and the power of big data more than ever before to overcome this global health issue^32^. This public health crisis has been aggravated by concomitant economic, humanitarian crises, and notable social and health disparity effects^66,67^. Now more than two years since the declaration of this pandemic and despite unprecedented planetary networks/initiatives, dedicated mega scale budgets, the intensive use of big data in drug/vaccines development, and the waves of seemingly effective vaccine, the ready access to vaccines is still a struggle in many parts of the world^17^. As mentioned above government dissemination strategies faltered still leaving today large portions of the population to be immunized and some countries of the world lagging well behind others. A balanced strategy of high biotechnology solutions and those other areas that affect socio-economic determinants that impact healthcare of the general population would have been well-served to meet the challenge of combatting this pandemic. Given the rapid mutation rate of SARS-CoV-2, the slow and not well-planned dissemination strategy may contribute to extending the pandemic as opposed to being the hopeful cure to end it. In contrast to individual-level management of the COVID-19 pandemic, it is noteworthy that population-level interventions mainly those targeting the socio-economic determinants of health would have the most impactful and cost-effective outcomes on flattening the pandemic curve^41,68^. In the interim, governments and societies have immediately sought refuge in social and lifestyle choices to alleviate the burden of the pandemic and to flatten the uprising infection curves until herd immunity of some sort is reached, i.e., a population-based approach to alleviate the impact. Lockdown, isolation of confirmed cases, quarantine of suspected infected and/or contacted individuals, social distancing, and the simple practice of wearing masks have been among the most effective social measures. Indeed, surges have been related to relaxing of these practices. These actions, together with lifestyle commitments as facemask wearing, frequent hand washing, healthy diet, exercise, and adequate sleep are considered as key tools to reduce the virus’ spread and flatten the epidemiological curve (Fig. 2).

## Conclusion

Despite big data and digital technologies’ intensive use during this global health crisis, they were quite helpful in the pandemic management (monitoring, surveillance, detection, prediction) and prevention measures^69–72^. Although some laudable initiatives have developed vaccines against SARS-CoV-2^73^, there is still speculation about their time frame, safety, and effectiveness of future remedies. It seems overly ambitious of the expectations (time frame, levels) for omics and big data to achieve their full potential in health and life sciences in general. Therefore, the current “omics and big data” solution in CHS, which undeniably offers potential benefits, should only be part of a larger and more comprehensive strategy. There needs to be more effort on holistic approaches that include health disparities, social determinants, and lifestyle choices to improve the quality of life. Social and economic systems should rethink cost/benefit analyses to determine the most effective ways to improve healthcare. While omics and digital technologies will have a substantial impact in healthcare and should be pursued, interventions, such as socio-economic determinants, that impact the greater population still will likely have more impact on CHS and must be part of our 21st century healthcare system.

### Reporting Summary

Further information on research design is available in the Nature Research Reporting Summary linked to this article.

## Supplementary information

Reporting Summary Checklist

## Data Availability

No datasets were generated or analyzed during the current study.
